# Gaussian Belief Propagation for Solving Network Utility Maximization with Delivery Contracts

**DOI:** 10.3390/e21070708

**Published:** 2019-07-19

**Authors:** Shengbin Liao, Jianyong Sun

**Affiliations:** 1 National Engineering Center for E-Learning, Huazhong Normal University, Wuhan 430079, China; 2 The National Engineering Laboratory for Educational Big Data Technology, Huazhong Normal University, Wuhan 430079, China; 3 The National Engineering Laboratory for Big Data Analytics and The School of Mathematics and Statistics, Xi’an Jiaotong University, Xi’an 710049, China

**Keywords:** network utility maximization, delivery contracts, Gaussian belief propagation, distributed algorithms

## Abstract

Classical network utility maximization (NUM) models fail to capture network dynamics, which are of increasing importance for modeling network behaviors. In this paper, we consider the NUM with delivery contracts, which are constraints to the classical model to describe network dynamics. This paper investigates a method to distributively solve the given problem. We first transform the problem into an equivalent model of linear equations by dual decomposition theory, and then use Gaussian belief propagation algorithm to solve the equivalent issue distributively. The proposed algorithm has faster convergence speed than the existing first-order methods and distributed Newton method. Experimental results have demonstrated the effectiveness of our proposed approach.

## 1. Introduction

Since the publication of the seminal paper [1] by Kelly et al., the framework of network utility maximization (NUM) has received a great deal of interest in the past two decades, which has been developed into a mathematical theory of network architectures [2]. Many important network design and resource allocation problems can be formulated as a NUM model. The utility concept, originally proposed in economics, is used to measure the satisfaction degree of a consumer for a good or service. In the basic NUM model, the utility of a network user is defined as a function of its data rate. The goal of network system is designed to maximize the overall utility of all the users in the network.

Consider a network with *L* links and *R* users, where each link *l* has a capacity of $c_{l}$ bps. Let L be the set of all links and a route *r* is a non-empty subset of L, let R be the set of possible routes, and associate a route *r* with a user *r* (or a data source *r*), i.e., L={1,2,…,L} and R={1,2,…,R}. Set $A_{lr}=1$ if l∈r, so that the link *l* lies on route *r*, and set $A_{lr}=0$ otherwise. This defines a 0-1 routing matrix $A=(A_{lr},l\in L,r\in R)$.

Suppose that if a rate $x_{r}$ is allocated to user *r* then this has utility $U_{r}\left(x_{r}\right)$ to the user. Assume that the utility $U_{r}\left(x_{r}\right)$ is increasing, strictly concave, and continuously differentiable over the range $x_{r}\geq 0$. Let $U=(U_{r}\left(x_{r}\right),r\in R)$ and $C=(c_{l},l\in L)$. Under this model, the network seeks a rate allocation $x=(x_{r},r\in R)$ which solves the following optimization problem [3].

(1) $$\begin{array}{cc} \max & \sum_{r\in R}U_{r}\left(x_{r}\right)\\ \mathrm{subject}\mathrm{to} & Ax\leq C\\ & \;x\geq 0 \end{array}$$

However, the basic NUM model (1) does not consider the network dynamics such as time-varying link capacities and user demands for quality of service (QoS). In this paper, we investigate a dynamic NUM model with QoS constraints, i.e., the model is time-varying over time *t*, which takes values in the set of time indices T={=1,2,…,T}. The model was first introduced in [4] as following

(2) $$\begin{array}{cc} \max & \sum_{t=1}^{T}\sum_{r=1}^{R}U_{r}^{t}\left(x_{r}^{t}\right)\\ \mathrm{subject}\mathrm{to} & A^{t}x^{t}\leq C^{t}\;\forall t\in T\\ & B_{r}x_{r}\geq q_{r}\;\forall r\in R\\ & \;x^{t}\geq 0\;\forall t\in T \end{array}$$

where $x_{r}^{t}$ denotes the source rate for user *r* at time step *t*, $x^{t}=\left(x_{r}^{t},r\in R\right)$ is the rate vector of all users at time step *t* and $x_{r}=\left(x_{r}^{t},t\in T\right)$ is the source rate allocation for user *r*. The second constraint in model (2) is the QoS constraints or delivery contracts. For each user, a delivery contract is the required minimal flow to be delivered over some particular time interval. Assume user *r* has $k_{r}$ delivery contracts and $q_{r}\in R^{k_{r}}$ is the associated contract quantity amounts. A contract is active at time index *t* if *t* is in the time interval of the contract. We can define a 0-1 matrix $B_{r}\in R^{k_{r}\times T}$ to represent the delivery contract indicator matrix by setting the ${\left(B_{r}\right)}_{kt}=1$ if the *k*th contract of user r is active at time *t*, and setting ${\left(B_{r}\right)}_{kt}=0$ otherwise. Thus, the QoS constraints that all delivery contracts are met can be given by $B_{r}x_{r}\geq q_{r}\;\forall r\in R$, i.e., the second constraint in model (2).

In the dynamic NUM model (2), utility function $U_{r}^{t}$, route matrix $A^{t}$ and link capacity $C^{t}$ are all dependent on the time index *t*, this means they are all possibly time-varying. There are many different ways to solve the problem (2), such as interior-point methods [5] and primary-dual algorithms [6]. The interior-point methods are efficient for solving the problem (2); however, they are centralized algorithms. The primary-dual algorithms are decentralized, but they suffer from slow convergence speed. In this paper, we concentrate on the issue of designing a distributed algorithm with fast convergence speed for solving the problem (2).

Only a few studies so far have investigated the problem (2) [4,7]. The goal of these works are similar to us. In [4], the authors presented a distributed primary-dual algorithm for solving the problem (2) based on dual decomposition and first-order methods. However, their work suffers from slow convergence speed. Of particular relevance to our work is [7], where a distributed Newton-type algorithm has been developed for solving the dynamic NUM model (2). Unlike these works, we use Gaussian belief propagation (GaBP) algorithm [8] to compute the Newton step and obtain an efficient distributed algorithm for solving the problem (2).

Our proposed solution is a *three-step method* for addressing the given issue. The first step is to obtain the optimality conditions for solving the problem (2) by introducing slack variables. The first step is similar to that approach used in the primary-dual algorithms for solving the problem (2). However, we do not adopt the primary-dual algorithms to solve the problem, which suffer from slow convergence speed.

The second step is to transform the obtained optimality conditions into an inference problem in a probabilistic graph model describing a certain Gaussian distribution with unknown parameters, which equal to optimal solutions for the problem (2). The method used in the second step transfers an algebraic problem into a probabilistic inference problem, which was first raised in [9].

The third step is to use the GaBP algorithm to evaluate distributively the parameter values of the Gaussian distribution. Essentially the GaBP algorithm is used to compute the Newton step in a primary-dual interior-point method [10]. This is similar to the work in [11]. However, this work does not consider the delivery contracts and is the special case of our work.

The outline of this paper is as follows. We first discuss the background and related work in Section 2, then present our method in Section 3. Section 4 provides experimental results and a discussion. Finally, Section 5 concludes this paper.

## 2. Background and Related Work

Before we present our idea, we first introduce a basic distributed optimization algorithm [3] which solves the model (1). Our work belongs to extensions of their works to dynamic model.

### 2.1. Basic Primary-Dual Algorithm

Low et al. present in [3] the following basic distributed optimization algorithm for solving the model (1).

The Lagrangian dual function for problem (1) is

(3) $$\begin{array}{c} D\left(\mu \right)\;\;=\max_{x_{r}\geq 0}\left\{\sum_{r\in R}U_{r}\left(x_{r}\right)-\mu^{Tr}\left(Ax-C\right)\right\}\\ \;\;\;=\max_{x_{r}\geq 0}\left\{\sum_{r\in R}U_{r}\left(x_{r}\right)-x_{r}\sum_{l\in L}A_{lr}\mu_{l}\right\}+\mu^{Tr}C \end{array}$$

where $\mu =(\mu_{l},l\in L)$ is a vector of Lagrange multipliers and $\mu^{Tr}$ denotes the transpose of the vector μ. Here, the second equality follows due to the definition of the matrix A. Thus, the dual problem for primary problem (1) is

(4) $$\begin{array}{cc} \min_{\mu \geq 0} & D(\mu ) \end{array}$$

In the dual formulation, Lagrange multiplier $\mu_{l}$ can be interpreted as congestion price on link *l*. A key observation from Equation (3) is that sources can compute their optimal rate individually, based on the total congestion price $\sum_{l\in L}A_{lr}\mu_{l}$, using the following source rate algorithm

(5) $$x_{r}=\arg\;\max_{x_{r}\geq 0}\left\{\sum_{r\in R}U_{r}\left(x_{r}\right)-x_{r}\sum_{l\in L}A_{lr}\mu_{l}\right\}$$

To solve the dual problem (4), one can use the following projected gradient method

(6) $$\mu_{l}\left(t+1\right)={\left[\mu_{l}\left(t\right)-\alpha \left(t\right)\left(c_{l}-\sum_{l\in L}A_{lr}x_{r}\right)\right]}^{+}$$

where α(t) is a positive scalar stepsize, and ${\left[a\right]}^{+}$ denotes the projection of *a* onto the set $R^{+}$ of non-negative real numbers.

According to the duality theory [12] and the assumption that the utility $U_{r}\left(x_{r}\right)$ is increasing, strictly concave and continuously differentiable over the range $x_{r}\geq 0$, the optimal solutions to both primary problem (1) and dual problem (4) can be found simultaneously by solving iteratively in Equations (5) and (6), respectively. This suggests treating the network links and the sources as processors in a distributed computation system to solve the primary problem (1) and the dual problem (4). The algorithm (5) and (6) is often referred to as the *basic primary-dual algorithm*. A large number of studies based on NUM framework belong to extensions of the basic primary-dual algorithm, the interested readers along this line please refer to [13].

### 2.2. Related Work

In the basic NUM model (1), the utility $U_{r}\left(x_{r}\right)$ of a network user *r* is defined as function of its data rate $x_{r}$, this means that all utility functions are separable. Due to the characteristic of separability of utility functions, a basic distributed NUM algorithm is derived to maximize aggregate user utility by the dual decomposition theory [12]. Along this way, so many extended NUM models and resultant distributed algorithms have been proposed for network architectural design, cross-layer optimization and resource allocation in wireless as well as wireline networks [14,15,16,17,18,19]. There are some works which studied the extended NUM models with the non-strictly concave or non-concave utility functions such as in [20,21,22,23]. When the utility functions are not strictly concave, the subgradient method is usually used to solve the dual problems instead of the gradient method in the *basic primary-dual algorithm*. If the utility functions are not concave, the extented duality method [24] can be used to construct distributed algorithms.

The design of the utility functions depends on applications of NUM problem. NUM-based approaches have been explored in different applications. Based on NUM, Liu et al. [25] presented a distributed and adaptive solution that jointly computes the data collection rates for each node and finds the schedule transmissions for rechargeable sensor networks. The concept of Water flow Driven Sensor Networks was introduced for leakage and contamination monitoring based on NUM [26]. Sadagopan et al. [27] use NUM approach constructing an energy balance tree in sensor networks, where each sensor node’s utility depends on the selection of its parent node. There exist some challenges to define utility functions based on performance metrics of different applications. The relationship between performance metrics and utility functions please refer to [28].

All the above works considered the static NUM models. Dynamic NUM models also belong to the extension of the basic NUM model. In [29], the authors presented a dynamic NUM model in adversarial environments. Their work focuses on the tradeoff between total queue length and utility regret. However, in this paper, we concentrate on the issue of devising a distributed algorithm to solve a dynamic NUM. In [30], the authors proposed a dynamic NUM model with time-varying fading channels. However, the utility functions and route matrix in their work are fixed. Moreover, their work focuses on the convergence behavior and tracking errors of the iterative primary-dual scaled gradient algorithm. Parametric network utility maximization model was presented in [31]. If the parameters are regarded as time steps, their model is equivalent to ours. However, their work concentrates on the tracking of algorithm trajectory by using a pathfollowing method on the parametric optimization problem [32].

The works in [4,7] are particular relevance to this work. The dynamic NUM with delivery contracts was first proposed in [4], and the authors presented a distributed primary-dual algorithm to solve the problem. The distributed primary-dual algorithm provided in [4] is based on dual decomposition theory, which is similar to the *basic primary-dual algorithm*. These primary-dual algorithms usually suffer from the slow rate of convergence [33,34].

The work in [7] also investigates the same model with ours in this paper. They proposed a distributed Newton method for solving the given problem. Their distributed algorithm obtained fast convergence speed compared with the distributed primary-dual algorithms. the method in [7] approximates the Newton direction at each iteration by using the matrix splitting technique. However, our method in this paper uses GaBP algorithm to evaluate the Newton direction.

Our proposed algorithm is a kind of primary-dual interior-point method. The primary-dual interior-point method was used for solving the NUM problem in [35]; however, the proposed algorithm is not decentralized. The work in [11] provided a distributed algorithm for solving a NUM by using the GaBP algorithm to compute the Newton direction. This is similar to our work. However, their model is static, and our model includes delivery contracts. Their work can be regarded as a special case of our work.

## 3. Our Method

In this section, we develop a *three-step method* to solve distributively the dynamic NUM problem (2). Before we present our idea, we first introduce some notations to simplify the model (2). let $x={\left(x_{1}^{Tr},x_{2}^{Tr},\ldots ,x_{R}^{Tr}\right)}^{Tr}$ and $C={\left(C_{1}^{Tr},C_{2}^{Tr},\ldots ,C_{T}^{Tr}\right)}^{Tr}$ be the rate vector of users and the capacity vector of links respectively, where $a^{Tr}$ is the transpose of the vector *a*. let matrix *A* denote the corresponding routing matrix for all time steps given as

$$A=\left(\begin{array}{cccc} A^{1} & 0 & \ldots & 0\\ 0 & A^{2} & \ldots & 0\\ \ldots & \ldots & \ldots & \ldots \\ 0 & 0 & \ldots & A^{T} \end{array}\right)$$

Then we can write down the first constraint of the dynamic NUM problem (2) as Ax≤C.

Similarly, let $q={\left(q_{1}^{Tr},q_{2}^{Tr},\ldots ,q_{R}^{Tr}\right)}^{Tr}$ be the contract quantity vector of users, and the matrix *B* denote the delivery contract matrix for all users given as

$$B=\left(\begin{array}{cccc} B_{1} & 0 & \ldots & 0\\ 0 & B_{2} & \ldots & 0\\ \ldots & \ldots & \ldots & \ldots \\ 0 & 0 & \ldots & B_{R} \end{array}\right)$$

Thus, the second constraint of the dynamic NUM problem (2) can be written as Bx≥q. Let *X* be the rate matrix with entries $\left(x_{r}^{t},r\in R,t\in T\right)$, define $U\left(X\right)=\sum_{t=1}^{T}\sum_{r=1}^{R}U_{r}^{t}\left(x_{r}^{t}\right)$

Equivalently, we can transform the dynamic NUM problem (2) as following,

(7) $$\begin{array}{cc} \max & U(X)\\ \mathrm{subject}\mathrm{to} & Ax\leq C\\ & Bx\geq q\\ & \;x\geq 0 \end{array}$$

Next, we will present our method to solve the problem (7).

### 3.1. Optimality Conditions

The Lagrangian associated with the NUM problem (7) is

(8) $$L\left(x_{r}^{t};\lambda ,\mu ,\alpha \right)=U\left(X\right)-\lambda^{Tr}\left(Ax-C\right)+\mu^{Tr}\left(Bx-q\right)+\alpha^{Tr}x$$

where λ, μ and α are Lagrange multiplier vectors which are associated the inequality constraints in the NUM problem (7). Therefore, the dual function is given by

$$D\left(\lambda ,\mu ,\alpha \right)=\max_{x_{r}\geq 0}\;L\left(x_{r}^{t};\lambda ,\mu ,\alpha \right)$$

Thus, the dual problem for model (7) is given by

(9) $$\min_{\lambda \geq 0,\mu \geq 0,\alpha \geq 0}\;D\left(\lambda ,\mu ,\alpha \right)$$

Assume ${\hat{x}}_{r}^{t}$, ($\hat{\lambda },\hat{\mu },\hat{\alpha }$) are the optimal solutions of the primary problem (7) and dual problem (9), according to the Karush-Kuhn-Tucker (KKT) conditions [12], we can obtain the optimality conditions as following,

(10) $$\begin{array}{c} -\nabla U\left(\hat{X}\right)+A^{Tr}\hat{\lambda }-B^{Tr}\hat{\mu }-\hat{\alpha }=0\\ \mathrm{diag}\left(\hat{\lambda }\right)\left(C-A\hat{x}\right)=0\\ \mathrm{diag}\left(\hat{\mu }\right)\left(B\hat{x}-q\right))=0\\ \mathrm{diag}\left(\hat{\alpha }\right)\hat{x}=0 \end{array}$$

where **diag**(·) denotes a diagonal matrix formed from its vector argument.

### 3.2. Inference Problem

We can modify the optimality conditions (10) and apply the primary-dual interior-point method on the modified optimality conditions for solving the primary problem (7) and dual problem (9) in an iterative manner with the given error of the duality gap [12]. The modification is parametrized by a parameter *k* as [35],

(11) $$\begin{array}{c} -\nabla U\left(X\right)+A^{Tr}\lambda -B^{Tr}\mu -\alpha =0\\ \mathrm{diag}\left(\lambda \right)\left(C-Ax\right)=\left(\frac{1}{k}\right)1\\ \mathrm{diag}\left(\mu \right)\left(Bx-q\right))=\left(\frac{1}{k}\right)1\\ \mathrm{diag}\left(\alpha \right)x=\left(\frac{1}{k}\right)1 \end{array}$$

where k≥0 is a parameter. We know from (11) that the modified optimality conditions approximate the optimality conditions as k→∞ and different values of *k* set the different accuracies of the approximation. We can compactly write the modified optimality conditions as following,

$$r_{t}\left(x,\lambda ,\mu ,\alpha \right)=\left[\begin{array}{c} -\nabla U\left(X\right)+A^{Tr}\lambda -B^{Tr}\mu -\alpha \\ \mathrm{diag}\left(\lambda \right)\left(C-Ax\right)-\left(\frac{1}{k}\right)1\\ \mathrm{diag}\left(\mu \right)\left(Bx-q\right))-\left(\frac{1}{k}\right)1\\ \mathrm{diag}\left(\alpha \right)x-\left(\frac{1}{k}\right)1 \end{array}\right]=0$$

The search direction of the primary-dual interior-point method is the Newton step for solving the modified optimality conditions $r_{t}\left(x,\lambda ,\mu ,\alpha \right)=0$. If $y={\left(x,\lambda ,\mu ,\alpha \right)}^{Tr}$ is the current point, the Newton step $\Delta y={\left(\Delta x,\Delta \lambda ,\Delta \mu ,\Delta \alpha \right)}^{Tr}$, then we have,

$$r\left(y+\Delta y\right)\approx r_{t}\left(y\right)+r_{t}^{\prime }\left(y\right)\Delta y=0,$$

where $r_{t}^{\prime }\left(y\right)$ denotes the derivative of $r_{t}\left(y\right)$. The above equation means

(12) $$-r_{t}\left(x,\lambda ,\mu ,\alpha \right)=\left[\begin{array}{cccc} -\nabla^{2}U\left(X\right) & A^{Tr} & -B^{Tr} & -I\\ -\mathrm{diag}(\lambda )A & \mathrm{diag}(C-Ax) & 0 & 0\\ \mathrm{diag}(\mu )B & 0 & \mathrm{diag}(Bx-q)) & 0\\ \mathrm{diag}(\alpha ) & 0 & 0 & \mathrm{diag}(x) \end{array}\right]\left[\begin{array}{c} \Delta x\\ \Delta \lambda \\ \Delta \mu \\ \Delta \alpha \end{array}\right]$$

Searching the Newton step by Equation (12) is the main computational bottleneck in the primary-dual interior-point method. However, in this paper, we do not directly calculate Newton’s direction by Equation (12). We transform the problem solving the liner Equation (12) into a probabilistic inference which can be computed based on GaBP. We first transfer the matrix in the right side of Equation (12) into a symmetric matrix by multiplying $r_{t}\left(x,\lambda ,\mu ,\alpha \right)$ a factor (1,−1/λ,−1/μ,−1/α) as following,

(13) $$\begin{array}{c} -{\hat{r}}_{t}\left(x,\lambda ,\mu ,\alpha \right)=\left[\begin{array}{cccc} -\nabla^{2}U\left(X\right) & A^{Tr} & -B^{Tr} & -I\\ A & -\mathrm{diag}(C-Ax)/\lambda & 0 & 0\\ -B & 0 & -\mathrm{diag}(Bx-q)/\mu & 0\\ -I & 0 & 0 & -\mathrm{diag}(x)/\alpha \end{array}\right]\left[\begin{array}{c} \Delta x\\ \Delta \lambda \\ \Delta \mu \\ \Delta \alpha \end{array}\right]\\ =A\left[\begin{array}{c} \Delta x\\ \Delta \lambda \\ \Delta \mu \\ \Delta \alpha \end{array}\right] \end{array}$$

where ${\hat{r}}_{t}\left(x,\lambda ,\mu ,\alpha \right)$ and A are defined as,

$${\hat{r}}_{t}\left(x,\lambda ,\mu ,\alpha \right)=r_{t}\left(x,\lambda ,\mu ,\alpha \right)\cdot {\left(1,-1/\lambda ,-1/\mu ,-1/\alpha \right)}^{Tr}=\left[\begin{array}{c} -\nabla U\left(X\right)+A^{Tr}\lambda -B^{Tr}\mu -\alpha \\ -\left(C-Ax\right)+\left(\frac{\lambda }{k}\right)1\\ -\left(Bx-q\right))+\left(\frac{\mu }{k}\right)1\\ -x+(\frac{\alpha }{k})1 \end{array}\right]$$

and

$$A=\left[\begin{array}{cccc} -\nabla^{2}U\left(X\right) & A^{Tr} & -B^{Tr} & -I\\ A & -\mathrm{diag}(C-Ax)/\lambda & 0 & 0\\ -B & 0 & -\mathrm{diag}(Bx-q)/\mu & 0\\ -I & 0 & 0 & -\mathrm{diag}(x)/\alpha \end{array}\right]$$

For notational simplicity, let $b=-{\hat{r}}_{t}\left(x,\lambda ,\mu ,\alpha \right)$, $w={\left(\Delta x,\Delta \lambda ,\Delta \mu ,\Delta \alpha \right)}^{Tr}$, we can write the Equation (13) as,

(14) Aw=b

Therefore, the Equation (14) for computing the Newton step is a system of linear equations with a symmetric coefficient matrix, which can be efficiently solved by using the GaBP algorithm [9]. We can define an undirected probabilistic graphical model G=(W,E), where W is a set of nodes which consist of the variables of linear Equation (14), and E is a set of edges which are determined by the non-zero entries of the coefficient matrix A. Given the matrix A and vector *b*, we can build up a Gaussian density function p(w)∼ exp$\left(-\frac{1}{2}w^{Tr}Aw+b^{Tr}w\right)$, which corresponds to the probabilistic graph G. Let $M=TR+TL+\sum_{r=1}^{R}Tk_{r}+TR$ be the dimension of the vector *b* (or vector *w*) (refer to the original model (2), we know that the number of the objective functions is TR, the capacity constraints are TL, the constraints of the delivery contracts are $\sum_{r=1}^{R}Tk_{r}$, and the non-negativity constraints are TR. Therefore, the dimension of the vector *b* is $TR+TL+\sum_{r=1}^{R}Tk_{r}+TR$). The probabilistic graph G has edge potentials (or compatibility functions) ψ and self-potentials (or evidence) ϕ. These graph potentials are determined by the following pairwise factorization of Gaussian distribution,

(15) $$p\left(w\right)\propto \prod_{i=1}^{M}\phi_{i}\left(w_{i}\right)\prod_{\left\{i,j\right\}}\psi_{ij}\left(w_{i},w_{j}\right),$$

resulting in $\phi_{i}\left(w_{i}\right)≐$exp$\left(-\frac{1}{2}A_{ii}w_{i}^{2}+b_{i}w_{i}\right)$ and $\psi_{ij}\left(w_{i},w_{j}\right)≐$exp$\left(-\frac{1}{2}w_{i}A_{ij}w_{j}\right)$. Using this probabilistic graph, we can transform the problem of solving the linear Equation (14) from the algebraic domain to a parameter estimation problem in the domain of probabilistic inference, as stated in the following theorem [9].

**Theorem** **1.** The computation of the solution vector $w^{*}=A^{-1}b$ is identical to the inference of the vector marginal means $\theta ≐\{\theta_{1},\ldots ,\theta_{R}\}$ over the graph G with the associated joint Gaussian probability density function $p\left(w\right)∼N\left(\theta ,A^{-1}\right)$.

**Proof.**  See Appendix A. □

The above theory shows that if we can distributively evaluate the mean of the Gaussian distribution p(w), then we can use the primary-dual interior-point method to distributively solve the primary problem (7) and dual problem (9). The next section will present the method for solving the mean of the inference problem (15) based on GaBP algorithm.

### 3.3. Parameter Evaluation Based on GaBP

Belief propagation is a kind of local message-passing algorithm and has been found to have excellent performance in many applications [36]. GaBP is a special case of the belief propagation algorithm, in which the underlying distributions are Gaussian. According to the statements in above section, in order to solve the linear equation problem (14) we need to infer the marginal densities $p(w_{i})$, which must also be Gaussian, i.e.,

$$p\left(w_{i}\right)∼N\left(\theta_{i}={\left\{A^{-1}b\right\}}_{i},\;P_{i}^{-1}={\left\{A^{-1}\right\}}_{ii}\right)$$

where $\theta_{i}$ and $P_{i}$ are the marginal mean and inverse variance (also known as the precision), respectively. Let N(i) be the set of all the nodes neighboring the node *i* (excluding node *i*). The set N(i)\j includes all the nodes in the set of N(i) except node *j*. The following Algorithm 1 provides the GaBP algorithm update rules for inferring the mean $\theta_{i}$.

**Algorithm 1:** GaBP Algorithm
Step 0 Initialization:Set a convergence threshold ϵ, $P_{ki}=0$ and $\theta_{ki}=0,\forall k\in N\left(i\right)$. Compute $P_{ii}=A_{ii}$ and $\theta_{ii}=b_{i}/A_{ii}$.Step 1 Iteration:Propagate the messages $P_{ki}$ and $\theta_{ki},\forall k\in N\left(i\right)$. Compute $P_{ij}=-A_{ij}^{2}/\left(P_{ii}+\sum_{k\in N(i)\backslash j}P_{ki}\right),\theta_{ij}=\left(P_{ii}\theta_{ii}+\sum_{k\in N(i)\backslash j}P_{ki}\theta_{ki}\right)/A_{ij}$.Step 2 Convergence check:If the message $P_{ij}$ and $\theta_{ij}$ do not converge, return to Step 1, else, go to Step 3.Step 3 Inference:Compute the marginal means $\theta_{i}=\left(P_{ii}\theta_{ii}+\sum_{k\in N(i)}P_{ki}\theta_{ki}\right)/\left(\left(P_{ii}+\sum_{k\in N(i)}P_{ki}\right)\right)$.Step 4 Output:Output the solution $w_{i}=\theta_{i}$.


## 4. Experiments and Analysis

### 4.1. Experimental Settings

In this section, we justify empirically the effectiveness of the proposed algorithm and compare the performance with the classical primary-dual algorithm and the primary-dual interior-point method. The classical primary-dual algorithm is based on dual decomposition. The primary-dual interior-point method used here is an iterative method for solving approximately a Newton system, which is usually called a truncated Newton primary-dual interior-point method [37].

We consider a network which has 100 flows and 200 links, and all of the utility functions are set to logarithmic functions, i.e., $U_{r}^{t}\left(x_{r}^{t}\right)=log\left(x_{r}^{t}\right)$, which are the most widely used in NUM problems [2]. The network was randomly generated and similar to that used in [34,35], this means that we need generate the link capacities and the routing matrix. The link capacities are chosen independently from a uniform distribution on [0.1, 1] and all of the required minimal flows to be delivered over different time intervals are set to 0.1, and the elements of the routing matrix *A* are generated randomly and independently, so that the average route length is 6 links. The time index *T* and all stepsizes are set to 10 and 0.001, respectively. After the network was generated, our proposed algorithm, primary-dual algorithm, and truncated Newton algorithm are performed once on it, respectively. The experimental results and comparisons are provided in the next section.

### 4.2. Experimental Results

**Figure 1:**
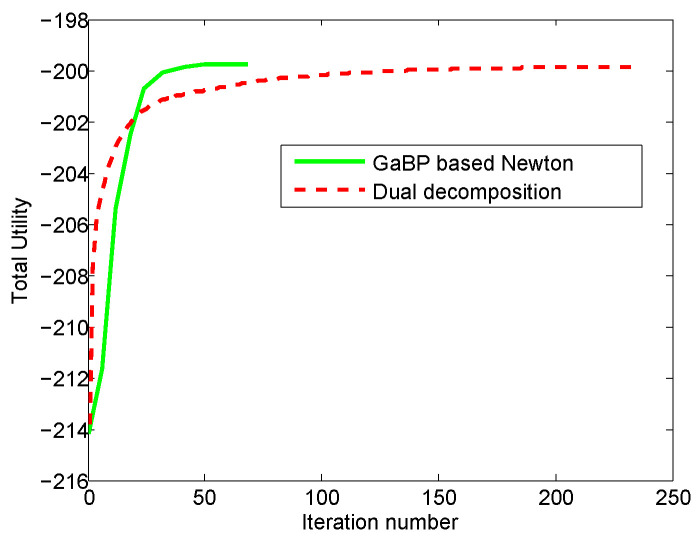
The convergence comparison of the proposed method and the dual decomposition algorithm. The green curve denotes the convergence speed of total utility for our proposed algorithm which is a distributed Newton method based on GaBP, and the red curve denotes the convergence speed of total utility for the dual decomposition algorithm which is a distributed primary-dual algorithm.

**Figure 2:**
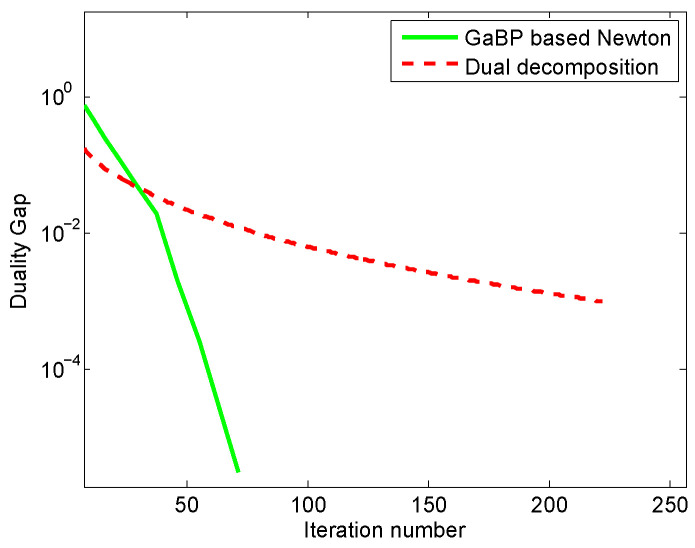
The duality gap comparison of the proposed method and the dual decomposition algorithm. The green curve denotes the estimation errors between the primary function and dual function versus iteration number for our proposed method, and the red curve denotes the estimation errors between the primary function and dual function versus iteration number for the dual decomposition algorithm.

We first evaluate the convergence of the proposed algorithm and compare with the classical primary-dual algorithm, these two algorithms are all distributed. Figure 1 shows the convergence curves of total utilities and Figure 2 provides the duality gap or corresponding residual values between the primary function and dual function.

**Figure 3:**
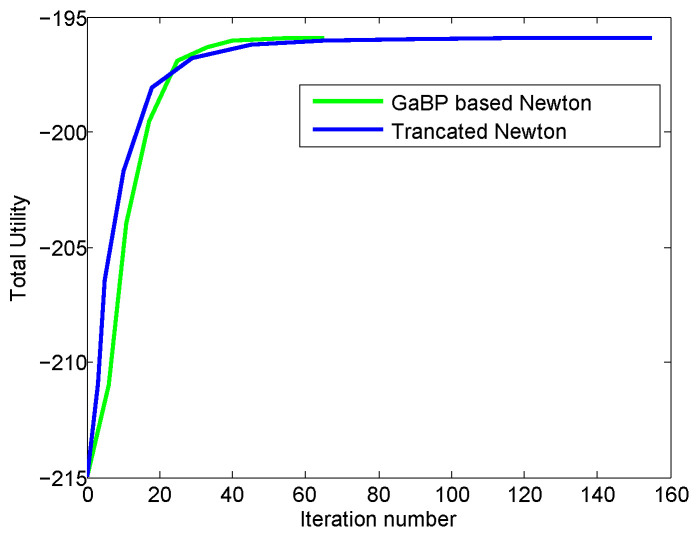
The convergence comparison of the proposed method and the truncated Newton method. The green curve denotes the convergence speed of total utility for our proposed algorithm which is a distributed Newton method based on GaBP, and the blue curve denotes the convergence speed of total utility for the truncated Newton method which is a centralized algorithm.

**Figure 4:**
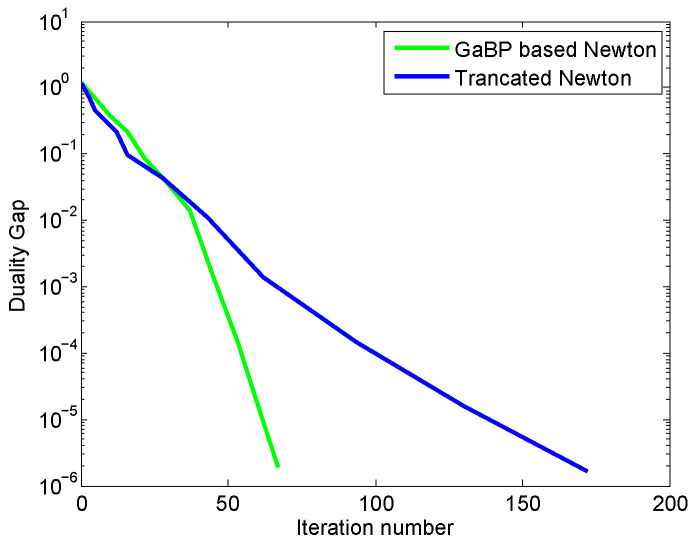
The duality gap comparison of the proposed method and the truncated Newton method. The green curve denotes the estimation errors between the primary function and dual function versus iteration number for our proposed method, and the blue curve denotes the estimation errors between the primary function and dual function versus iteration number for the truncated Newton algorithm.

We also compare the performance of our proposed method with the truncated Newton method which has achieved a very fast convergence speed and very good accuracy for solving nonlinear equation system. However, the truncated Newton method is centralized. Figure 3 and Figure 4 show the convergence curves of total utilities and the duality gap curves versus iteration number for our proposed method and the truncated Newton method, respectively.

Our proposed method uses GaBP algorithm to compute the Newton and the truncated Newton method adopts the preconditioned conjugate gradient (PCG) algorithm [38] for computing the Newton step. While the above Figure 3 and Figure 4 provide the performance comparison in term of the Newton steps, we also give the comparison of the iteration count in each Newton step for these two algorithms in Table 1 and Table 2. Table 1 and Table 2 are the experimental results for two networks which have 100 flows and 200 links, and 500 flows and 1000 links, respectively.

### 4.3. Experiment Analysis

#### 4.3.1. Analysis and Comparison with a Distributed Method

We will analyze the experimental results in terms of convergence speed and solution accuracy. From Figure 1, we can see that our proposed algorithm and the dual decomposition algorithm can converge to the optimal value Within a certain range of errors. However, the convergence speed of our method is much faster than the dual decomposition algorithm.

The duality gap between the primary function and the dual function depicts the accuracy of the obtained solution. For a convex optimization problem, the primary variables and dual variables will eventually approach the optimal solution as the duality gap tends to zero. Naturally, we expect that our proposed algorithm will obtain a smaller duality gap. From Figure 2, we can see that the duality gap achieved by our proposed algorithm is smaller than that obtained by the dual decomposition method.

#### 4.3.2. Analysis and Comparison with a Centralized Approach

The truncated Newton method is a centralized approach, which is an efficient primary-dual interior-point method and achieves good performance in many optimization problems [39]. We gave the performance comparison for our proposed method and the truncated Newton method in this section.

From Figure 3, we can see that the convergence speed of our proposed method is very fast, which is slightly faster than the truncated Newton method. This means that both methods had comparable convergence speed. However, as specified before, our proposed approach is distributed, while the truncated Newton method is centralized.

Figure 4 provides the accuracy comparison of the solutions obtained by both methods. From Figure 4, we can see that our proposed method has a smaller duality gap than that achieved by the truncated Newton method. This means that although both methods had comparable convergence rate, the accuracy of the solution obtained by our method is better than that achieved by the truncated Newton method.

As these two methods computed the Newton steps based on GaBP and PCG algorithms respectively, we also compared the iteration count for each Newton step. From Table 1 and Table 2, we can see that the iteration count of GaBP algorithm is smaller than that required by the PCG algorithm except for the first few Newton steps. Moreover, the total iteration count of GaBP algorithm is also smaller than that achieved by the PCG algorithm.

Another advantage for GaBP algorithm is that as the Newton step number increases, the iteration count for each Newton step tends to a stable value. However, the iteration count required by PCG algorithm always increases; moreover, the increased magnitudes grows bigger as the scale of the network grows.

## 5. Conclusions

We propose a three-step method for distributively solving network utility maximization with delivery contracts (or dynamic NUM). This paper first obtained the optimality conditions for solving the dynamic NUM problem by dual decomposition theory. Then we transform the problem for searching the Newton step in solving the optimality conditions into a probabilistic inference. Finally, GaBP algorithm was used to compute the probabilistic inference.

NUM problems are usually solved by means of the classical primary-dual algorithm, which is used as the benchmark algorithm for testing the effectiveness of our proposed method. By comparing and analyzing the experimental results, we can reach a conclusion that the proposed method is effective in convergence speed and solution accuracy compared with the classical primary-dual algorithm.

Our proposed method belongs to distributed primary-dual interior-point methods. Therefore, we also compared the performance of our proposed method with the primary-dual interior-point method based on PCG, which had achieved a very fast convergence speed and very good accuracy for solving nonlinear equation problems. The experimental results also validated the effectiveness of our proposed method.

## Figures and Tables

**Table 1:** Experimental results of the iteration count for each Newton step in a small network.

Newton Step Number	GaBP	PCG
1	6	3
2	6	2
3	6	2
4	7	5
5	9	9
6	10	12
7	12	13
8	15	22
9	14	29
10	13	34
11		43
total	98	174

**Table 2:** Experimental results of the iteration count for each Newton step in a bigger network.

Newton Step Number	GaBP	PCG
1	6	2
2	5	4
3	5	8
4	6	3
5	7	16
6	7	20
7	7	36
8	7	64
9		101
total	50	253

## Abbreviations

The following abbreviations are used in this manuscript:

NUM	Network utility maximization
GaBP	Gaussian belief propagation
QoS	Quality of service
KKT	Karush-Kuhn-Tucker
PCG	preconditioned conjugate gradient
${\left(\cdot \right)}^{Tr}$	The transpose of a matrix or a vector

## Appendix A

**Proof** **of** **Theorem** **1.** Solving the linear equation system Aw=b is equivalent to maximizing the quadratic form $-\frac{1}{2}w^{Tr}Aw+b^{Tr}w$, which is further equivalent to finding the maximal value of the exponential function exp$\left(-\frac{1}{2}w^{Tr}Aw+b^{Tr}w\right)$. Next, we can define the joint Gaussian probability density function as follows:

(A1) $$p\left(w\right)≐Z^{-1}\exp\left(-\frac{1}{2}w^{Tr}Aw+b^{Tr}w\right)$$

where Z is a normalization factor to make p(w) to be a probability distribution. Let $\theta ≐A^{-1}b$, we can rewrite the joint Gaussian probability density function as following,

(A2) $$\begin{array}{c} \begin{array}{c} p\left(w\right)=Z^{-1}\exp\left(\frac{1}{2}\theta^{Tr}A\theta \right)\exp\left(-\frac{1}{2}w^{Tr}Aw+\theta^{Tr}Aw-\frac{1}{2}\theta^{Tr}A\theta \right)\\ =\xi \exp(-\frac{1}{2}{\left(w-\theta \right)}^{Tr}A\left(w-\theta \right))\\ =N(\theta ,A^{-1}) \end{array} \end{array}$$

where $\xi =Z^{-1}\exp\left(\frac{1}{2}\theta^{Tr}A\theta \right)$ is the new normalization factor after the distribution p(w) has been rewritten. From the above Equation (A2), we see that the mean vector of the Gaussian distribution p(w) defined by Equation (A1) is θ, which is equal to the our target solution $w^{*}=A^{-1}b$. This proves Theorem 1. □
